# Subacute Bronchitis

**Published:** 1889-02

**Authors:** A. McNeil


### SUBACUTE BRONCHITIS.

October 21st.—Captain Erickson, æt. fifty, just returned from a voyage to Alaska. He was short-handed, and had to be exposed very much. For last three months has had a frightful cough, so constant as to leave him but little sleep, and took away his appetite, so that he was much reduced in flesh and strength. It was so constant that he could not give me any modalities. The irritation to cough was a constant tickling in the upper third of the chest on both sides. The cough was attended by a great deal of dyspnœa, much anxiety; coughed a long time and then raised a little mucus; some soreness in the chest. He has a hernia, and every cough presses down on the sac and testicles.

Gave him Zinc-met.$^{30}$, one powder. This was followed by prompt relief until November 20th, when the cough became worse. Gave Zinc$^{205}$, one powder, but no relief followed, and I gave him Rhus, Lach., Arsen., Phos., and Iodium, but he went from bad to worse till he scarcely slept or ate; was feverish; anguish and dyspnœa increased till I became apprehensive that it would end in chronic bronchitis and death. The symptoms still remaining the same as first helped by Zinc., I concluded to return to it, and gave two powders of the 500th on December 4th. Next day a trifle better, and so till now he is almost well. Did the Zinc.$^{205}$ possess any virtue? I think not. And its worthlessness nearly cost a human life.

